# An Immunohistochemical Evaluation of Tumor-Associated Glycans and Mucins as Targets for Molecular Imaging of Pancreatic Ductal Adenocarcinoma

**DOI:** 10.3390/cancers13225777

**Published:** 2021-11-18

**Authors:** Ruben D. Houvast, Kira Thijse, Jesse V. Groen, JiaXin Chua, Mireille Vankemmelbeke, Lindy G. Durrant, J. Sven D. Mieog, Bert A. Bonsing, Alexander L. Vahrmeijer, Peter J. K. Kuppen, A. Stijn L. P. Crobach, Cornelis F. M. Sier

**Affiliations:** 1 Department of Surgery, Leiden University Medical Center, 2333 ZA Leiden, The Netherlands; R.D.Houvast@lumc.nl (R.D.H.); Kira_2000@live.nl (K.T.); J.V.Groen@lumc.nl (J.V.G.); J.S.D.Mieog@lumc.nl (J.S.D.M.); B.A.Bonsing@lumc.nl (B.A.B.); A.L.Vahrmeijer@lumc.nl (A.L.V.); P.J.K.Kuppen@lumc.nl (P.J.K.K.); 2 Scancell Limited, University of Nottingham Biodiscovery Institute, University Park, Nottingham NG7 2RD, UK; jiaxin.mymab@gmail.com (J.C.); MireilleVankemmelbeke@scancell.co.uk (M.V.); Lindy.Durrant@nottingham.ac.uk (L.G.D.); 3 Division of Cancer and Stem Cells, School of Medicine, University of Nottingham Biodiscovery Institute, University Park, Nottingham NG7 2RD, UK; 4 Department of Pathology, Leiden University Medical Center, 2333 ZA Leiden, The Netherlands; A.S.L.P.Crobach@lumc.nl; 5 Percuros BV, 2333 CL Leiden, The Netherlands

**Keywords:** pancreatic cancer, aberrant glycosylation, carbohydrates, mucins, biomarkers

## Abstract

**Simple Summary:**

Distinguishing pancreatic cancer from healthy tissue before and during surgery can be enhanced by using molecular tracers directed at molecules on tumor cells allowing high-contrast visualization of tumor tissue, eventually improving diagnosis and surgical removal. Albeit sugar molecules and proteins carrying a large amount of sugars-mucins- have gained significant interest as tumor-specific targets, their relative presence on structures surrounding tumor tissues and lymph node metastases is unknown. The current study shows that the presence of several, but not all, investigated sugar molecules and mucins on pancreatic cancer cells is higher compared to surrounding tissues. Moreover, given their abundance on tumor cells in lymph nodes and their absence on normal lymph nodes, all investigated targets are high-potential targets for visualization of lymph node metastases. This study paves the way for the development of molecular tracers against the targets evaluated herein to allow improvement of pancreatic cancer treatment.

**Abstract:**

Targeted molecular imaging may overcome current challenges in the preoperative and intraoperative delineation of pancreatic ductal adenocarcinoma (PDAC). Tumor-associated glycans Le^a/c/x^, sdi-Le^a^, sLe^a^, sLe^x^, sTn as well as mucin-1 (MUC1) and mucin-5AC (MU5AC) have gained significant interest as targets for PDAC imaging. To evaluate their PDAC molecular imaging potential, biomarker expression was determined using immunohistochemistry on PDAC, (surrounding) chronic pancreatitis (CP), healthy pancreatic, duodenum, positive (LN^+^) and negative lymph node (LN^−^) tissues, and quantified using a semi-automated digital image analysis workflow. Positive expression on PDAC tissues was found on 83% for Le^a/c/x^, 94% for sdi-Le^a^, 98% for sLe^a^, 90% for sLe^x^, 88% for sTn, 96% for MUC1 and 67% for MUC5AC, where all were not affected by the application of neoadjuvant therapy. Compared to PDAC, all biomarkers were significantly lower expressed on CP, healthy pancreatic and duodenal tissues, except for sTn and MUC1, which showed a strong expression on duodenum (sTn tumor:duodenum ratio: 0.6, *p* < 0.0001) and healthy pancreatic tissues (MUC1 tumor:pancreas ratio: 1.0, *p* > 0.9999), respectively. All biomarkers are suitable targets for correct identification of LN^+^, as well as the distinction of LN^+^ from LN^−^ tissues. To conclude, this study paves the way for the development and evaluation of Le^a/c/x^-, sdi-Le^a^-, sLe^a^-, sLe^x^- and MUC5AC-specific tracers for molecular imaging of PDAC imaging and their subsequent introduction into the clinic.

## 1. Introduction

Pancreatic ductal adenocarcinoma (PDAC) is the seventh leading cause of cancer-related mortality in the Western world, with a dismal 5-year survival of only 9% [1]. As 80–90% of patients present with locally advanced or metastatic disease, radical surgical resection, which is the only curative therapy, is often not feasible. Extensive preoperative imaging using endoscopic ultrasound (EUS), magnetic resonance imaging (MRI) and positron emission tomography/computed tomography (PET/CT) is crucial for accurate selection and stratification of patients for surgery. Nevertheless, 20–47% of patients who qualify for surgery present with an irresectable disease at the time of surgery [2,3], whereas R1 (microscopic residual disease) resections are reported in up to 80% of patients, both of which are associated with worse overall survival [4,5,6]. On the other hand, approximately 7% of resections for suspected pancreatic cancer are performed for benign diseases, such as chronic pancreatitis (CP) [7]. Considering the abundance of desmoplasia in both PDAC and CP, which may be further induced by the application of neoadjuvant therapy (NAT), distinguishing malignant from healthy or benign tissue is challenging in both a preoperative and real-time intraoperative setting [8,9,10]. By facilitating high-contrast visualization of tumor cells, targeted molecular imaging may play a key role in overcoming these challenges, potentially avoiding resection for benign and irresectable disease, while simultaneously aiming to increase radical resection rates in resectable patients.

Within the continuing search for novel targets for molecular imaging, tumor-associated glycans and mucins have gained significant interest (reviewed in [11]). In cancer, many proteins and lipids are aberrantly glycosylated, which results in the appearance of truncated *O*-glycans, such as sialyl-Thomsen-Nouveau (sTn) and Lewis glycans, such as sialyl-Lewis^a^ (sLe^a^) and sialyl-Lewis^x^ (sLe^x^), Lewis^a/c/x^ (Le^a/c/x)^, sialyl-di-Lewis^a^ (sdi-Le^a^) and related glyco-epitopes [12,13,14,15]. Some of these structures, such as sLe^a^ and sLe^x^, are involved in tumor progression, both directly and indirectly by applying conformational changes to their carrier protein [16,17].

In reference to glycans, mucins, which are high-molecular-weight proteins that are extensively coated with *O*-glycans, seem interesting tumor-specific targets based on their high expression on tumor tissues, low abundance in healthy tissues and pivotal roles in carcinogenesis [18,19] Especially, transmembrane mucin-1 (MUC1) and secreted mucin-5AC (MUC5AC), which are both, directly and indirectly, involved in tumor progression via their truncated sTn glycans, are considered promising targets for PDAC targeting [18]. As a result of mucin overexpression, tumor-associated glycans become strongly amplified on the outermost layer of multiple proteins simultaneously, making them a set of high-potential molecular imaging targets with advantages for targeting beyond proteins [11,20]. Although the aforementioned tumor-associated *O*-glycans and mucins are strongly expressed on pancreatic cancers cells, their relative expression on (surrounding) chronic pancreatitis as well as on healthy pancreas and duodenum and metastatic lymph nodes, which defines their molecular imaging suitability, is underexplored. 

Therefore, the current study aims to evaluate and compare the potential of tumor-associated glycans Le^a/c/x^, sdi-Le^a^, sLe^a^, sLe^x^ and sTn, and mucins MUC1 and MUC5AC for molecular imaging of PDAC using a semi-automated, machine learning-based digital image analysis workflow. 

## 2. Materials and Methods

### 2.1. Patient and Tissue Selection

Medical records and pathology reports from patients who underwent pancreatic resection in the Leiden University Medical Center (LUMC) between August 2011 and July 2020 were retrospectively reviewed. Patients older than 18 years diagnosed with PDAC or CP were considered suitable for inclusion in the study. Representative formalin-fixed paraffin-embedded tissue blocks containing PDAC, CP, healthy pancreatic, healthy duodenum, LN^+^ and LN^−^ tissues were obtained from the Pancreas Biobank of the LUMC. All tissue samples were assessed by a hepatopancreaticobiliary pathologist (A.S.L.P.C.) before inclusion in the study. Both peritumoral pancreatitis and primary CP tissues were categorized as CP. Clinicopathological data were retrospectively collected from hospital records. R1 resection was defined as the presence of tumor cells at ≤1 mm from the surgical margin. Pathological T (pT) and pathological N (pN) stages were defined according to the 8th edition of the AJCC/UICC TNM staging system for pancreatic cancer. The study protocol was approved by the Gastroenterology Biobank Review Committee (protocol reference: 2020-16) and local medical ethical review committee (protocol reference: B20.052). The research was conducted in accordance with the Dutch code of conduct for responsible use of human tissue in medical research. Tissue samples and patient data were used anonymously and in compliance with the Declaration of Helsinki (1964).

### 2.2. Monoclonal Antibodies and Reagents

Le^a/c/x^, sdi-Le^a^, sLe^a^, sLe^x^, sTn, MUC1 and MUC5AC were selected based on their expected specificity for PDAC. The primary and secondary mAbs and other reagents are listed in Table S1.

### 2.3. Immunohistochemistry (IHC)

The 4-μm-thick formalin-fixed paraffin-embedded tissue sections were placed on glass slides. The sections were deparaffinized in xylene for 15 min, rehydrated in a series of 100%, 50%, 25% ethanol dilutions and rinsed in demineralized water. Next, endogenous peroxidase was blocked for 20 min using 0.3% hydrogen peroxide in demineralized water. Antigen retrieval was subsequently performed as described in Table S1. After cooling in phosphate-buffered saline (PBS, pH 7.4), sections were incubated overnight in a humidified chamber at room temperature with 150 μL primary antibody using a predetermined optimal dilution (see Table S1). Next, slides were washed three times in PBS for 5 min and incubated with appropriate secondary antibodies, followed by an additional washing step. Staining was visualized through incubation with 3,3-diaminobenzidine tetrahydrochloride solution (DAB, K3468, Agilent Technologies, Inc., Santa Clara, CA, USA) for 10 min at room temperature. Sections were then counterstained with Mayer’s hematoxylin solution (Sigma-Aldrich, Saint Louis, MO, USA). After dehydration in an incubator for 1 h at 37 °C, slides were mounted with Pertex (Leica Microsystems, Wetzlar, Germany).

### 2.4. Semi-Automated Imaging Analysis

Whole slide images of tissue sections were captured using a PANNORAMIC^®^ 250 Flash III DX scanner (3DHISTECH Ltd., Budapest, Hungary) and imported into QuPath v.0.2.3 [21]. All tissue slides were scanned using similar settings to exclude variability during image analysis. A detailed description and graphic representation of the object classifier training, validation and semi-automated image analysis workflow is included in the Supplementary Materials. Briefly, random forest object classifiers for PDAC, pancreatic (healthy pancreas and CP), healthy duodenal, positive lymph node (LN^+^) and negative lymph node (LN^−^) tissue classes were built for each biomarker [22]. QuPath parameters used for automated cell detection are listed in Table S2. Object classifiers were trained until they provided detection of their respective cell type with a sensitivity, specificity, positive predictive value (PPV), negative predictive value (NPV) and accuracy of ≥85%, as depicted in Figure S1. Next, tissue class-, biomarker-specific scripts allowing semi-automated cell detection, segmentation, object classifier application and classification of DAB staining intensity were generated as shown in Figure S2. DAB staining intensity was classified as negative, low (1+), moderate (2+) or strong (3+). Next, PDAC, CP, healthy pancreas, healthy duodenum, LN^+^ and LN^−^ regions were then annotated on the full cohort by a pathologist (A.S.L.P.C.), after which the respective script was run (Figure S2). Staining was quantified using the H-score (formula: 1 × (% cells 1+) + 2 × (% cells 2+) + 3 × (% cells 3+), range: 0–300). Immunohistochemical staining with an H-score ≥ 51 was regarded positive [23]. 

### 2.5. Statistical Analysis

Statistical analysis and graph generation were performed using IBM SPSS statistics (version 25, IBM Corporation, Somer, NY, USA) and GraphPad Prism (version 8, GraphPad Software, La Jolla, CA, USA). Baseline characteristics between groups were compared using a Chi-square test for categorical data, an unpaired t-test for normally distributed data or Mann–Whitney U test for nonparametric data. Mean H-scores were compared using one-way ANOVA with Bonferroni correction (≥3 groups) or an unpaired *t*-test (2 groups). Receiver operating characteristic (ROC) curves were drawn to calculate area under the curve (AUC) for LN^+^ vs. LN^−^ detection based on H-score. Differences with a *p*-value < 0.05 were considered statistically significant.

## 3. Results

### 3.1. Patient Characteristics

Tissues from 53 patients primarily diagnosed with PDAC and 9 patients diagnosed with CP were obtained. The clinicopathological data of this cohort are summarized in Table 1. Of the PDAC cohort, 22 patients received NAT, of which 15 patients received chemoradiotherapy and 7 patients received chemotherapy. NAT patients were significantly younger (*p* = 0.033) had significantly lower pN stages (*p* < 0.001), smaller tumors (*p* = 0.024) and lower serum CA19-9 levels (*p* = 0.007) compared to PDAC patients who did not receive NAT. Slides containing PDAC tissue were not available for 5 patients. In total, tissue blocks containing 48 PDAC, 28 CP, 31 healthy pancreatic, 10 healthy duodenal, 27 LN^+^ and 41 LN^−^ tissues derived of 62 patients (53 PDAC and 9 CP patients) were included in the study. 

### 3.2. Object Classifier Training and Validation

To prepare the scripts for semi-automated image analysis, thirty-five tissue class-, biomarker-specific object classifiers were trained and validated as described in the Supplementary Materials. Briefly, after extensive training, sensitivity, specificity, PPV, NPV and accuracy were above the predetermined threshold of 85% for all object classifiers separately, allowing highly accurate detection and classification of its cell type of interest (Table S3).

### 3.3. Biomarker Expression on PDAC, CP, Healthy Pancreatic and Duodenal Tissues

**Figure 1:**
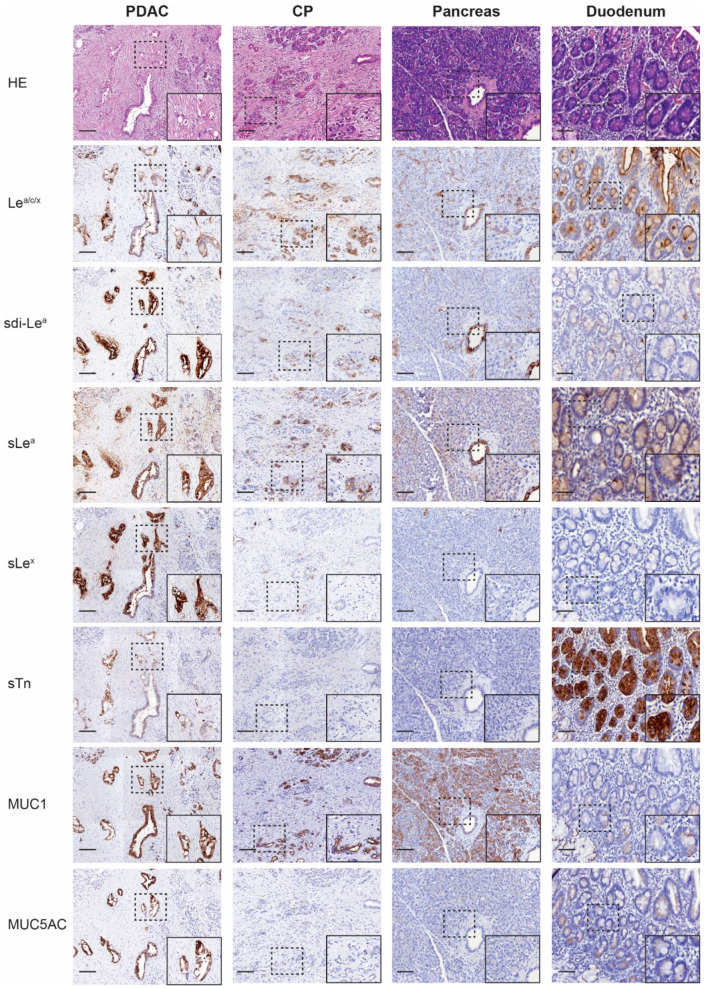
Representative (immuno)histochemical staining of HE, Le^a/c/x^, sdi-Le^a^, sLe^a^, sLe^x^, sTn, MUC1 and MUC5AC expression on PDAC, CP, pancreas and duodenum tissues. HE: hematoxylin-eosin, CP: chronic pancreatitis, PDAC: pancreatic ductal adenocarcinoma, Overview images and inserts are taken at 5× and 25× magnification, respectively. Scale bars represent 100 µM.

**Figure 2:**
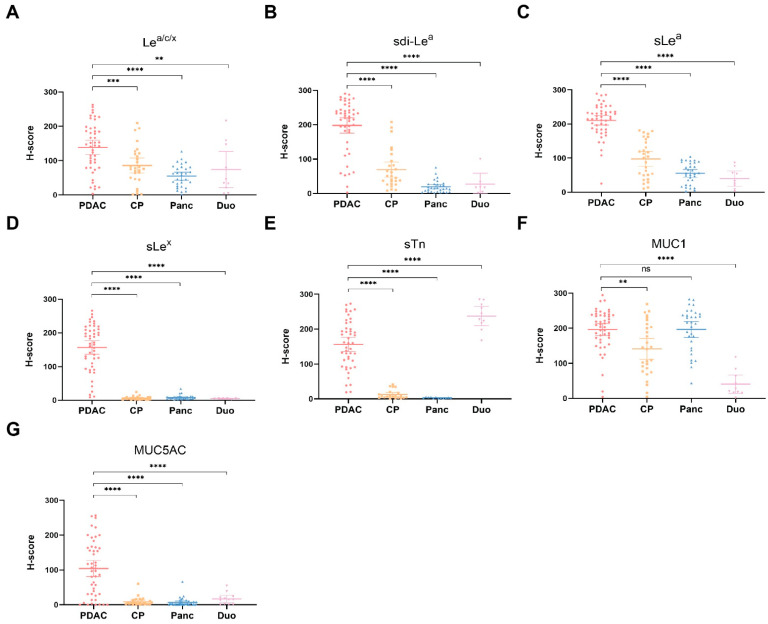
H-score scatter plots of immunohistochemical staining of (**A**) Le^a/c/x^; (**B**) sdi-Le^a^; (**C**) sLe^a^; (**D**) sLe^x^; (**E**) sTn; (**F**) MUC1; (**G**) MUC5AC expression on PDAC, CP, pancreas and duodenum tissues. Mean H-scores are represented by the horizontal line together with their error bars representing the 95% confidence interval. Within each tissue category, every dot represents immunohistochemical staining on one case. CP: chronic pancreatitis, Duo: duodenum, ns: not significant, Panc: pancreas, PDAC: pancreatic ductal adenocarcinoma, **: *p* < 0.01, ***: *p* < 0.001, ****: *p* < 0.0001.

The cohort was stained for Le^a/c/x^, sdi-Le^a^, sLe^a^, sLe^x^, sTn, MUC1 and MUC5AC (Figure 1), followed by semi-automated imaging analysis. H-scores scatter plots showing IHC staining of all biomarkers on PDAC, CP, healthy pancreatic and duodenal tissues are depicted in Figure 2.

Positive biomarker expression on PDAC tissues was found on 83% for Le^a/c/x^ (40/48), 94% for sdi-Le^a^ (45/48), 98% for sLe^a^ (47/48), 90% for sLe^x^ (43/48), 88% for sTn (42/48), 96% for MUC1 (46/48) and 67% for MUC5AC (32/48), as shown in Table 2. Categorized IHC staining distributions on PDAC tissues and biomarker expression for each PDAC case separately are represented in Table 3 and in heatmap format in Figure S3, respectively. All biomarkers were highly expressed on tumor tissues and showed a tumor-specific, membranous staining pattern of PDAC cells. Le^a/c/x^, sdi-Le^a^, sLe^a^ and sTn showed a more heterogenous staining distribution, while sLe^x^, MUC1 and MUC5AC staining was slightly more homogenous. Moreover, strong luminal staining was occasionally observed for Le^a/c/x^, sdi-Le^a^, sLe^a^ and sLe^x^, but not for MUC1 and MUC5AC. 

In CP, staining was homogenous and mainly located on acinar and ductal cells of the pancreas. Low to moderate staining was observed for Le^a/c/x^, sdi-Le^a^, sLe^a^ and MUC1, while sLe^x^, sTn and MUC5AC expression was virtually absent. For all biomarkers, expression in CP was significantly lower than in PDAC, although tumor:CP ratios of only 1.7 and 1.4 were observed for Le^a/c/x^ and MUC1, respectively (Table 2). 

Low to moderate Le^a/c/x^, sdi-Le^a^ and sLe^a^ expression was found in healthy acinar cells, while MUC1 was highly expressed. As for CP, expression in healthy pancreatic tissue was mainly located on acinar and ductal cells. sLe^x^, sTn and MUC5AC expression was virtually absent. Compared to PDAC, a significantly lower healthy pancreas expression was found for all biomarkers (*p* < 0.0001), except for MUC1 (tumor:pancreas ratio: 1.0, *p* > 0.9999). 

In healthy duodenal tissues, low to moderate expression of Le^a/c/x^, sdi-Le^a^, sLe^a^ and MUC1 on cells of the glandular epithelium was observed, in which Le^a/c/x^ expression was more abundant relative to sdi-Le^a^, sLe^a^ and MUC1. Moreover, strong sTn staining was observed. Of note, occasional staining of Brunner’s glands was present for sLe^a^, sLe^x^, sTn, MUC1, MUC5AC, but to a lesser extent for Le^a/c/x^ and sdi-Le^a^. Expression on healthy duodenal tissue was significantly lower compared to PDAC for all biomarkers (*p* < 0.0001), except for sTn (tumor:duodenum ratio: 0.6, *p* < 0.0001), as shown in Table 2.

### 3.4. Biomarker Expression on PDAC Tissues after NAT 

**Figure 3:**
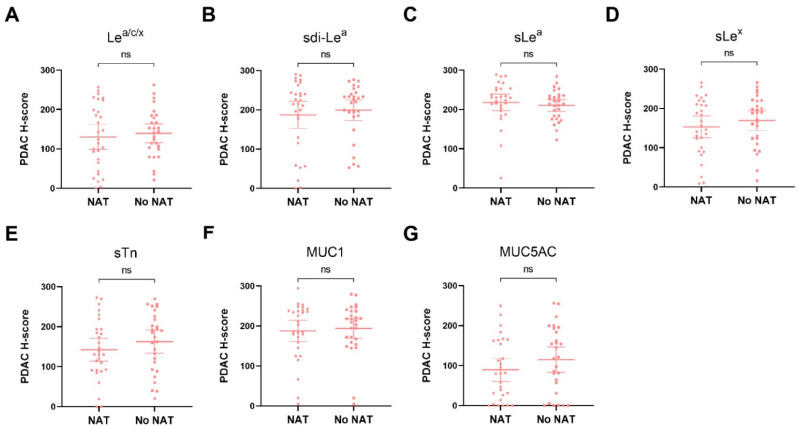
H-score scatter plots of immunohistochemical staining of (**A**) Le^a/c/x^; (**B**) sdi-Le^a^; (**C**) sLe^a^; (**D**) sLe^x^; (**E**) sTn; (**F**) MUC1; (**G**) MUC5AC expression on PDAC tissues of patients who received NAT or no NAT. Mean H-scores are represented by the horizontal line together with their error bars representing the 95% confidence interval. Each dot represents immunohistochemical staining on one case. NAT: neoadjuvant treatment, ns: not significant.

As we found that all biomarkers showed high expression on PDAC tissues, subgroup analyses were performed to study the effect of NAT on biomarker expression on PDAC tissues. H-score scatter plots showing biomarker expression in NAT and no NAT patients are shown in Figure 3. Although sLe^x^, sTn and MUC5AC expression seemed slightly lower in the NAT group, no statistically significant differences in biomarker expression between NAT and no NAT patients were observed, suggesting that NAT does not influence the (over)expression of these biomarkers. 

### 3.5. Biomarker Co-Expression on PDAC Tissues

Biomarker co-expression on tumor tissues was analyzed to evaluate the potential added value of targeting two biomarkers simultaneously. The percentage of patients with positive expression of at least one biomarker along with the percentage of cases with biomarker co-expression are shown in Table 4. Although co-expression was present in the majority of patients, virtually all patients expressed at least one of two biomarkers of any panel, with the least-performing biomarker combination being sTn-MUC5AC that was, alone and or combined, expressed in 90% of PDAC tissues. The highest co-expression panel was sLe^a^ and MUC1, which were simultaneously expressed in 94% of patients.

### 3.6. Detection of Lymph Node Metastases

**Figure 4:**
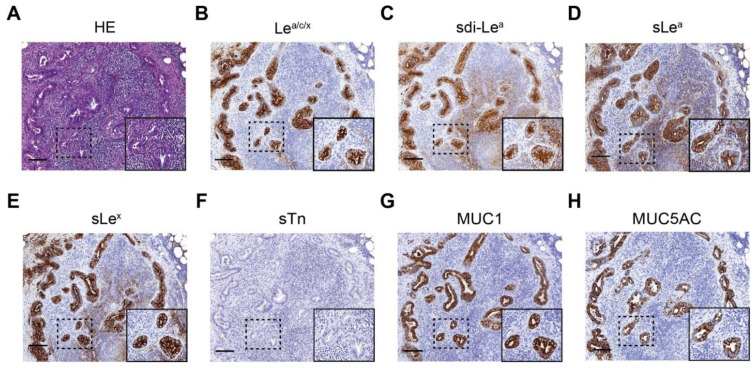
Representative images of (immune)histochemical staining of (**A**) HE; (**B**) Le^a/c/x^; (**C**) sdi-Le^a^; (**D**) sLe^a^; (**E**) sLe^x^; (**F**) sTn; (**G**) MUC1; (**H**) MUC5AC expression on LN^+^ tissues of primary PDAC patients. Overview images and inserts are taken at 5× and 25× magnification, respectively. Scale bars represent 100 µM. HE: hematoxylin-eosin, LN^+^: positive lymph node.

**Figure 5:**
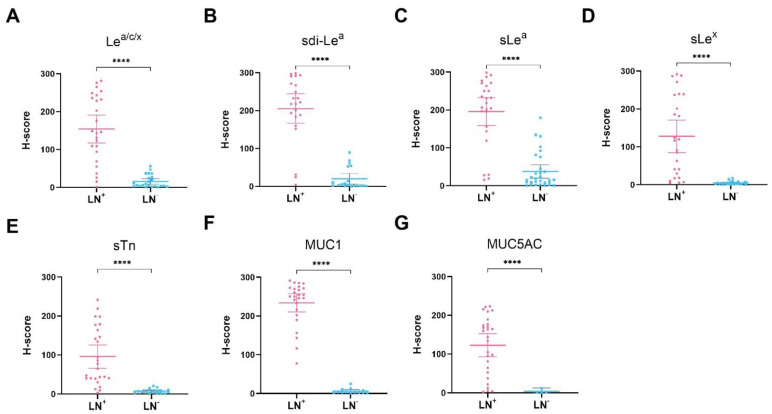
H-score scatter plots of immunohistochemical staining of (**A**) Le^a/c/x^; (**B**) sdi-Le^a^; (**C**) sLe^a^; (**D**) sLe^x^; (**E**) sTn; (**F**) MUC1; (**G**) MUC5AC expression on LN^+^ and LN^−^ tissues. Mean H-scores are represented by the horizontal line together with their error bars representing the 95% confidence interval. Within each tissue category, every dot represents immunohistochemical staining on one case. LN^+^: positive lymph node, LN^−^: negative lymph node, ****: *p* < 0.0001.

LN^+^ and LN^−^ tissues were stained to evaluate the biomarkers’ potential for identification of lymph node metastases in addition to primary PDAC lesions. Representative IHC images for biomarker expression on LN^+^ tissues are depicted in Figure 4, which shows that all biomarkers were highly expressed on PDAC cells in LN^+^ tissues. For LN^−^ tissues, biomarker expression was mostly absent, although low to moderate expression was occasionally observed for Le^a/c/x^, sdi-Le^a^ and sLe^a^. Despite the latter, mean LN^+^ expression was significantly higher compared to LN^−^ expression for all biomarkers (*p* < 0.0001), as shown in Figure 5. In addition, sensitivity, specificity, PPV, NPV and AUC for correct LN^+^ detection were calculated based on positive or negative biomarker expression on LN^+^ and LN^−^ tissues. Although sensitivity for LN^+^ detection was lower for sLe^x^ and sTn, Le^a/c/x^, sdi-Le^a^, sLe^a^, MUC1 and MUC5AC showed high LN^+^ identification potential, with limited false-positive and false-negative staining. Accuracy for identification of LN^+^ and LN^−^ tissues together was 90% for Le^a/c/x^, 81% for sdi-Le^a^, 81% for sLe^a^, 84% for sLe^x^, 81% for sTn, 97% for MUC1, and 91% for MUC5AC (Table 5).

## 4. Discussion

Through specific binding to and (real-time) visualization of tumor cells, targeted molecular imaging agents can play a key role in overcoming current challenges during diagnosis, resection, and monitoring of PDAC. In this study, we evaluated the potential of tumor-associated glycans Le^a/c/x^, sdi-Le^a^, sLe^a^, sLe^x^ and sTn, and mucins MUC1 and MUC5AC as a molecular imaging target for PDAC using a semi-automated, machine-learning-based image analysis workflow. Our results show that all biomarkers are highly expressed on PDAC cells. Importantly, subgroup analyses showed that biomarker expression was similar in patients who received NAT and patients who did not receive NAT, suggesting that NAT does not influence biomarker expression. This finding is particularly promising in view of the ever-increasing application of neoadjuvant chemoradiotherapy for PDAC and paves the way for PDAC targeting using these biomarkers in a clinically relevant setting [24]. We additionally showed that simultaneous targeting of two targets using, for instance, a bispecific tracer could be attractive in order to allow targeting of the entire PDAC population. High tumor:CP ratios were observed for all biomarkers, although tumor:CP ratios for MUC1 and Le^a/c/x^ were closer to 1 (1.4 and 1.7, respectively). In addition, high tumor:pancreas ratios were observed for all biomarkers, except for MUC1 (tumor:pancreas ratio 1.0). These results suggest that all biomarkers, besides MUC1, have a high potential to serve as molecular imaging targets to solve current challenges in the delineation of primary PDAC lesions from surrounding CP and healthy pancreatic tissue. We additionally evaluated biomarker expression on healthy duodenal tissues to evaluate their potential for delineating locally advanced primary pancreatic head carcinomas invading the duodenum, which can be present in 47–58% of patients [25,26]. In contrast to the other biomarkers, sTn’s abundant expression on healthy duodenal tissues limits its suitability for molecular imaging of primary PDAC invading the duodenum.

In addition to primary PDAC detection, both pre- and intraoperative imaging of lymph node metastases is pivotal for disease staging and monitoring [27,28]. Therefore, we evaluated the potential of the biomarker panel to detect lymph node metastases and found that all biomarkers are significantly upregulated on LN^+^ compared to LN^−^ tissues. All biomarkers showed a high detection potential for LN^+^ tissues and distinction of LN^+^ from LN^−^ tissues, which was comparable to the performance of established protein-based molecular imaging targets, such as CEACAM5, PSMA, avβ6 and uPAR, further strengthening their potential as molecular imaging targets [28,29]. 

Due to their tumor-specific (over)expression and excellent in vivo accessibility, tumor-associated glycans, which are present on the outermost layer of the cell membrane, are of particular interest for molecular imaging [11]. Several glycan-specific tracers were successfully evaluated for molecular imaging of PDAC in a preclinical setting, but only a few studies have described glycan-based imaging in a clinical context. For instance, ^89^Zr-DFO-HuMab-5B1 (MVT-2163), which targets sLe^a^ (more commonly known as CA19-9), was successfully evaluated in a phase 1 trial for PET imaging of PDAC and provided clear delineation of primary tumors and metastases, some of which were not identified using standard imaging modalities [30]. sLe^a^ is also employed as a serum biomarker for diagnosis and monitoring of PDAC within standard-of-care. However, despite its strong overexpression in PDAC, targeting of sLe^a^ in PDAC is limited by its presence in the healthy pancreas, CP and other benign pancreaticobiliary diseases, which is confirmed by the relatively low tumor:CP, tumor:pancreas and tumor:duodenum ratios found in the current study [31]. Noteworthy, we showed that sdi-Le^a^, which is a Lewis glycan structurally related to sLe^a^, had a more restricted expression on CP, healthy pancreas and duodenal tissues with similar PDAC expression, which strengthens the major potential of sdi-Le^a^ over sLe^a^ for specific PDAC targeting. 

Le^a/c/x^ and sdi-Le^a^ were recently described by Chua and Tivadar et al., respectively, showing high expression on PDAC tissues with low to moderate abundance on healthy tissues [13,15]. Once employed in vivo, the Le^a/c/x^-specific mAb FG88.2 subsequently displayed remarkable tumor targeting [13]. Recently, our group conducted a proof-of-concept evaluation of the chimeric (human/mouse) counterpart of the FG88.2 mAb, CH88.2, as a targeting moiety for fluorescence-guided surgery of colon carcinoma and PDAC. Conjugated to IRDye 800CW, the tracer allowed clear visualization of subcutaneous HT-29 (colon carcinoma) and BxPC-3 (PDAC) tumor xenografts using a clinical near-infrared fluorescence imaging system [32]. Although additional IHC exploration of expression on other gastrointestinal tumors along with their normal counterparts and metastases is required to evaluate the tracer employability beyond PDAC, the current findings strongly support previous data on FG88.2 staining, paving the way for a clinical translation of the tracer [13,15].

In addition to glycans, mucins, that are heavily coated with glycans, may form attractive targets for molecular imaging of PDAC due to their tumor-specific expression, some of which from the earliest in situ stage onward. Although in our study MUC1 seems to be a less suitable candidate for molecular imaging of PDAC, it should be noted that alternative conformational epitopes on MUC1, induced by the presence of (truncated) *O*-glycans, were described [33,34]. As their accessibility is dependent on conformational changes, induced by tumor-specific aberrant glycosylation, their expression on healthy tissues might be minimized, making them more attractive for tumor-specific targeting. For instance, the PAM4-reactive epitope, which is present on both MUC1 and MUC5AC, was shown to have a low abundance on healthy pancreatic and CP tissues, while expression on PDAC and pancreatic intraepithelial neoplasia (PanIN)-1A lesions onward was high [35,36]. Evaluation of PAM4-reactive epitope expression on the current cohort would be an interesting continuation in order to establish its potential as a PDAC imaging target, while simultaneously putting the current findings into perspective. 

A strong methodological point of the study is the inclusion of tissues derived from the entire PDAC context, i.e., the primary tumor, healthy/benign tissue counterparts, surrounding organs and metastatic and healthy lymph nodes, which is paramount for a complete and accurate biomarker comparison. Our semi-automated image analysis workflow provided highly accurate cell classification, allowing an objective, reproducible and precise evaluation of biomarker expression. In contrast, accurate manual scoring of heterogeneous biomarker stainings may be challenging and consequently suffers from both intraobserver and interobserver variability [37,38,39]. Moreover, to the best of our knowledge, this study is the first to evaluate the expression of the current biomarkers on both PDAC tissues of patients who received NAT and on metastatic PDAC lymph node tissues.

This study has some limitations. Application of the current QuPath workflow for this relatively small cohort is limited by its labor intensity and still does not avoid the involvement of a specialized pathologist. In addition, erroneous classification of out-of-focus tissue areas and staining artifacts, although mostly avoided during tissue area annotation, may further compromise accurate semi-automated scoring of digital images. Moreover, we cannot fully exclude that, particularly in patients that received NAT, residual tumor clusters in both primary resection and lymph node tissues were misclassified and subsequently annotated as non-tumorous. It should however be noted that considering manual scoring to be the gold standard may overlook the potential of machine learning-based algorithms to classify cells with superior accuracy relative to the human eye [40]. Furthermore, we feel that the benefits of the highly accurate, semi-automated scoring method, which is of high importance considering the heterogenicity of the observed staining patterns within a complex PDAC morphology, do outweigh the aforementioned disadvantages. 

This study identified Le^a/c/x^, sdi-Le^a^, sLe^a^, sLe^x^, and MUC5AC as high-potential targets for molecular imaging of PDAC. Future research into glycan- and mucin-targeted imaging should thus focus on the development and evaluation of clinically suitable tracers directed against these glycan and mucin targets. Secondly, although this study showed no difference in biomarker expression on PDAC tissues between NAT and no NAT patients, evaluating the correlation between biomarker expression on PDAC tissues before and after NAT, for instance by using fine-needle aspiration biopsies acquired before NAT, could strengthen the current finding that NAT does not influence biomarker expression. Thirdly, although this study demonstrates the potential of identifying LN^+^ tissues based on the expression of the evaluated biomarkers, future animal models with complex lymph node metastases are required to definitely establish a glycan or mucin-targeting tracer’s potential for metastatic lymph node detection. Altogether, this study provides a strong foundation for the development, characterization and preclinical evaluation of tumor-associated glycan- and mucin-specific molecular imaging agents for high-contrast delineation of PDAC. 

## 5. Conclusions

To conclude, our results show that particularly Le^a/c/x^, sdi-Le^a^, sLe^a^, sLe^x^ and MUC5AC are high-potential targets for molecular imaging of primary PDAC lesions, regardless of the application of NAT. Due to their strong abundance on duodenum and healthy pancreatic tissues, sTn and MUC1 were considered less suitable targets. All biomarkers are suitable targets for correct identification of LN^+^ as well as the distinction of LN^+^ from LN^−^ tissues. Through this study, we lay the groundwork for the development and evaluation of clinically suitable glycan- and mucin-specific tracers for molecular imaging of PDAC. 

## Figures and Tables

**Table 1:** Characteristics of PDAC patients (*n* = 53) and CP patients (*n* = 9) *. PDAC patients are categorized into NAT and no NAT patients. *p*-values represent differences between NAT and no NAT patients. CP: chronic pancreatitis, IQR: interquartile range, NA: not applicable, NAT: neoadjuvant therapy, PDAC: pancreatic ductal adenocarcinoma, SD: standard deviation.

Characteristic	Total PDAC (*n* = 53)	NAT (*n* = 22)	No NAT (*n* = 31)	*p*-Value	CP (*n* = 9)
Age, years, mean (SD)	64.7 (9.8)	61.3 (9.1)	67.1 (9.7)	0.033	53.5 (10.9)
Gender, *n* (%)					
Male	26 (49)	9 (41)	17 (55)	0.406	8 (89)
Female	27 (51)	13 (59)	14 (45)		1 (11)
Surgery type, *n* (%)					
Pancreaticoduodenectomy	41 (77)	16 (73)	25 (81)	0.632	4 (44)
Pancreatic corpus/tail resection	9 (17)	4 (18)	5 (16)		5 (56)
Total pancreatectomy	3 (6)	2 (9)	1 (3)		0 (0)
Tumor differentiation, *n* (%)					
Good	6 (11)	1 (5)	5 (16)	0.607	-
Moderate	12 (23)	1 (5)	11 (36)		-
Poor	18 (34)	4 (18)	14 (45)		-
Missing	17 (32)	16 (73)	1 (3)		-
Primary tumor, *n* (%)					
pT1	18 (34)	10 (46)	8 (26)	0.275	-
pT2	27 (51)	10 (46)	17 (55)		-
pT3	8 (15)	2 (9)	6 (19)		-
Regional lymph nodes, *n* (%)					
pN0	18 (34)	13 (59)	5 (16)	<0.001	-
pN1	21 (40)	9 (41)	12 (39)		-
pN2	14 (26)	0 (0)	14 (45)		-
Surgical margin status, *n* (%)					
R0	29 (55)	15 (68)	14 (45)	0.161	-
R1	24 (45)	7 (32)	17 (55)		-
NAT, *n* (%)					
No	31 (59)	0 (0)	31 (100)	-	8 (89)
Yes, chemoradiotherapy	15 (28)	15 (68)	0 (0)	-	0 (0)
Yes, chemotherapy	7 (13)	7 (32)	0 (0)	-	1 (11)
Tumor size, mm, mean (SD)	26 (13)	22 (11)	30 (13)	0.024	-
Serum CEA, µg/L, median (IQR)	3.2 (5.9)	3.2 (6.5)	3.5 (5.2)	0.349	-
Serum CA19-9, kU/L, median (IQR)	74.5 (377.5)	48.4 (69.7)	322.8 (371.6)	0.007	-

* Patients primarily diagnosed with CP are listed in the table as a separate cohort next to PDAC patients.

**Table 2:** Percentage of PDAC tissues with positive immunohistochemical staining (H-score ≥ 51 out of 300) and mean tumor:CP, tumor:pancreas and tumor:duodenum H-score ratios, along with the *p*-value of the H-score difference. CP: chronic pancreatitis, PDAC: pancreatic ductal adenocarcinoma.

Biomarker	PDAC Positive *n* (%)	Tumor: CP	*p*-Value	Tumor: Pancreas	*p*-Value	Tumor: Duodenum	*p*-Value
Le^a/c/x^	40 (83)	1.7	0.0010	2.5	<0.0001	1.9	0.0073
sdi-Le^a^	45 (94)	2.9	<0.0001	10.3	<0.0001	10.0	<0.0001
sLe^a^	47 (98)	2.2	<0.0001	3.8	<0.0001	5.9	<0.0001
sLe^x^	43 (90)	33.2	<0.0001	20.9	<0.0001	53.0	<0.0001
sTn	42 (88)	15.6	<0.0001	100.9	<0.0001	0.6	<0.0001
MUC1	46 (96)	1.4	0.0012	1.0	>0.9999	4.8	<0.0001
MUC5AC	32 (67)	11.5	<0.0001	13.6	<0.0001	5.6	<0.0001

**Table 3:** Distribution of biomarker expression on 48 PDAC tissues (*n* (%)). Expression was categorized as negative (H-score: 0–50), low (H score: 51–100), moderate (H-score: 101–200) or high (H-score 201–300). PDAC: pancreatic ductal adenocarcinoma.

PDAC Expression				
Biomarker	Negative *n* (%)	Low *n* (%)	Moderate *n* (%)	High *n* (%)
Le^a/c/x^	8 (17)	8 (17)	23 (48)	9 (19)
sdi-Le^a^	3 (6)	5 (10)	11 (23)	29 (60)
sLe^a^	1 (2)	0 (0)	15 (31)	32 (67)
sLe^x^	5 (10)	7 (15)	20 (42)	16 (33)
sTn	6 (13)	7 (15)	23 (48)	12 (25)
MUC1	2 (4)	1 (2)	18 (38)	27 (56)
MUC5AC	16 (33)	9 (19)	17 (35)	6 (13)

**Table 4:** Percentage of cases with positive expression for at least one of two biomarker combinations (panel: ≥1) along with the percentage of cases with expression of both biomarkers (panel: both). Immunohistochemical staining with an H-score of ≥51.0 was considered positive.

Biomarker	Panel	Le^a/c/x^ (%)	sdi-Le^a^ (%)	sLe^a^ (%)	sLe^x^ (%)	sTn (%)	MUC1 (%)	MUC5AC (%)
Le^a/c/x^	≥1 Both	-	-	-	-	-	-	-
sdi-Le^a^	≥1 Both	94 83	-	-	-	-	-	-
sLe^a^	≥1 Both	100 81	100 92	-	-	-	-	-
sLe^x^	≥1 Both	98 75	100 83	100 88	-	-	-	-
sTn	≥1 Both	100 71	100 81	100 85	98 79	-	-	-
MUC1	≥1 Both	100 79	100 90	100 94	100 85	100 83	-	-
MUC5AC	≥1 Both	96 54	96 65	98 67	96 60	90 65	96 67	-

**Table 5:** Biomarker sensitivity, specificity, PPV, NPV and accuracy along with the AUC and *p*-value for identification of LN^+^. Immunohistochemical staining with an H-score of ≥51.0 was considered positive. AUC: area under the curve, PPV: positive predictive value, NPV: negative predictive value, Sens.: sensitivity, Spec.: specificity, 95% CI: 95% confidence interval.

Biomarker	Sens. (%)	Spec. (%)	PPV (%)	NPV (%)	Accuracy (%)	AUC (95% CI)	*p*-Value
Le^a/c/x^	78	98	96	87	90	0.929 (0.846–1.000)	<0.0001
sdi-Le^a^	70	88	79	82	81	0.955 (0.896–1.000)	<0.0001
sLe^a^	78	83	75	85	81	0.927 (0.858–0.995)	<0.0001
sLe^x^	59	100	100	79	84	0.960 (0.913–1.000)	<0.0001
sTn	52	100	100	76	81	0.954 (0.894–1.000)	<0.0001
MUC1	93	100	100	95	97	1.000 (1.000–1.000)	<0.0001
MUC5AC	78	100	100	87	91	0.972 (0.912–1.000)	<0.0001

## Data Availability

The data presented in this study are available on request from the corresponding author. The data are not publicly available due to privacy reasons.

## Supplementary Materials

The following are available online at https://www.mdpi.com/article/10.3390/cancers13225777/s1. Figure S1: Graphical representation of biomarker training and validation workflow; Figure S2: QuPath images and semi-automated image analysis workflow; Figure S3: Heatmap of biomarker expression on PDAC tissues for each case separately. Table S1: Primary and secondary mAbs, clone, catalog number, provider, isotype and conditions used during IHC; Table S2: Automated cell detection parameters used in QuPath; Table S3: Mean ± SD object classifier sensitivity, specificity, PPV, NPV, accuracy for the detection of tumor, stromal, acinar, immune or glandular cells pooled from all biomarkers (*n* = 7).

## Abbreviations

95% CI: 95% Confidence Interval, AUC: Area Under the Curve, CP: Chronic Pancreatitis, DAB: 3,3′-Diaminobenzidine, EUS: Endoscopic Ultrasound, HE: Hematoxylin-Eosin, IHC: Immunohistochemistry, IQR: Interquartile Range, Le^a/c/x^: Lewis^a/c/x^, LN^+^: positive lymph node, LN^−^: negative lymph node, MRI: Magnetic Resonance Imaging, MUC1: Mucin-1, MUC5AC: Mucin-5AC, NAT: Neoadjuvant Therapy, NPV: Negative Predictive Value, PanIN: Pancreatic Intraepithelial Neoplasia, PBS: Phosphate-Buffered Saline, PDAC: Pancreatic Ductal Adenocarcinoma, PET/CT: Positron Emission Tomography/Computed Tomography, PPPD: Pylorus-Preserving Pancreatoduodenectomy, PPV: Positive Predictive Value, ROC: Receiver Operation Characteristic, ROI: Region-Of-Interest, SD: Standard Deviation, sdi-Le^a^: sialyl-di-Lewis^a^, Sens.: Sensitivity, sLe^a^: sialyl-Lewis^a^, sLe^x^: sialyl-Lewis^x^, Spec.: Specificity, sTn: sialyl-Thomsen-Nouveau.
