# Supplementary material for: An Immunohistochemical Evaluation of Tumor-Associated Glycans and Mucins as Targets for Molecular Imaging of Pancreatic Ductal Adenocarcinoma
Source: Cancers (Basel). 2021 Nov 18;13(22):5777. doi: 10.3390/cancers13225777 (PMC8616289; doi:10.3390/cancers13225777)
Supplement: Supplementary file 1 [file cancers-13-05777-s001.zip › cancers-1453224-supplementary.pdf]

cancers

MDPI

*Supplementary Materials*

# An Immunohistochemical Evaluation of Tumor-Associated Glycans and Mucins as Targets for Molecular Imaging of Pancreatic Ductal Adenocarcinoma

**Ruben D. Houvast, Kira Thijse, Jesse V. Groen, JiaXin Chua, Mireille Vankemmelbeke, Lindy G. Durrant, J. Sven D. Mieog, Bert A. Bonsing, Alexander L. Vahrmeijer, Peter J.K. Kuppen, A. Stijn L. P. Crobach, Cornelis F. M. Sier**

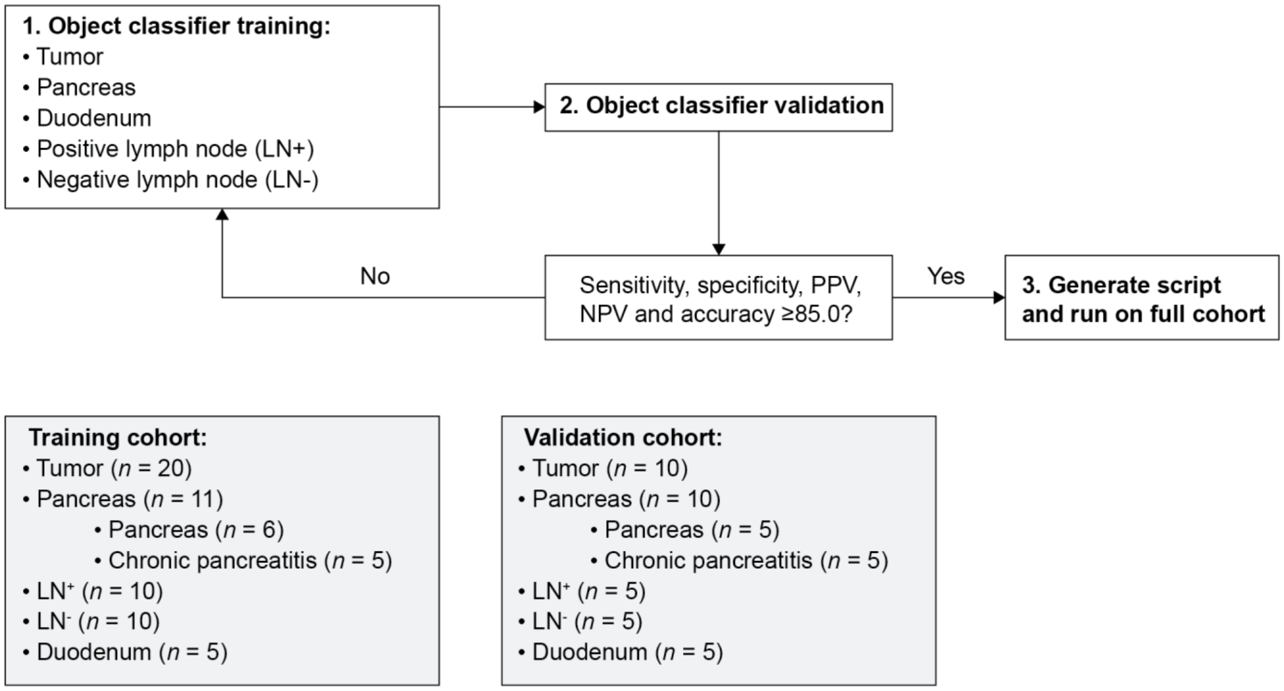


**Figure S1.** Graphical representation of biomarker training and validation workflow. LN+: positive lymph node, LN−: negative lymph node, PPV: positive predictive value, NPV: negative predictive value.

## *1.1. Object Classifier Training*

To allow artificial intelligence-based, semi-automated analysis of biomarker staining on tissues, extensive random forest object classifiers training was performed in QuPath v.0.2.3 using a randomly selected training set of 20 PDAC, 10 CP/pancreas, 5 duodenum, 10 LN+ and 10 LN− tissue slides per biomarker (Figure S1). First, automated cell detection, segmentation and smoothing (Radius: 25 µm, smooth within classes: true) were applied over annotated regions-of-interest (ROI). QuPath parameters used for automated cell detection are listed in Table S2. Next, tumor, acinar cell, immune cell, duodenal gland and stromal tissue areas were manually annotated. Tissue class-specific training data from the training set were pooled to generate a final tissue class-, biomarker-specific object classifier. In total, thirty-five object classifiers (five tissue classes for seven biomarkers) were built.

## *1.2. Object Classifier Validation*

To evaluate the object classifiers' performance, validation was performed using 10 PDAC, 5 healthy CP/pancreas, 5 LN+, 5 LN− and 5 duodenum slides that were not included in the training set. For each PDAC, CP/pancreas and duodenum slides, 10 random tumor, acinar or duodenal gland cell areas and 10 random stromal cell areas were manually annotated. For LN+ and LN− tissues, 5 tumor, immune and or stroma cell regions were annotated. All tissue areas solely contained the cell type of interest, allowing quantification of correct and incorrect cell detections after application of the object classifier. After automated cell detection, segmentation, smoothing and application of the corresponding object classifier, cell classifications and measurements within each ROI were exported followed by calculation of object

classifier performance indicators based on the number correctly and incorrectly detected cells within all annotated ROIs. Object classifiers with a sensitivity, specificity, positive predictive value (PPV), negative predictive value (NPV), accuracy of ≥ 85% were regarded sufficiently trained. Representative brightfield and QuPath mask images of each tissue class are depicted in Figure S2A–F. Mean object classifier sensitivity, specificity, PPV, NPV, accuracy and precision for the detection of tumor, stromal, acinar, immune or glandular cells from all biomarkers combined ($n$ = 7) are shown in Table S3.

### *1.3. Semi-Automated Image Analysis*

To prepare the scripts for semi-automated analysis of biomarker staining, cell intensity thresholds, 1+, 2+ and 3+ for weak, moderate and strong staining, respectively, were determined for each biomarker separately in a multidisciplinary meeting (Measurement: smoothed: 25 μm; cytoplasm: DAB OD mean) and included in the script. Next, tissue class-, biomarker-specific scripts allowing semi-automated cell detection, segmentation, object classifier application followed by cell intensity classification were generated (Figure S2A). For semi-automated image analysis, PDAC, CP, pancreas, duodenum, LN+ and LN- regions were annotated on the full cohort by a pathologist (A.S.L.P.C.). Since QuPath does not allow simultaneous application of multiple object classifiers within one project, annotated slides were exported to tissue class-specific projects. Next, the corresponding script was run, and cell measurement data were exported and analyzed. Biomarker staining was quantified automatically by QuPath using the H-score (formula: 1 × (% cells 1+) + 2 × (% cells 2+) + 3 × (% cells 3+), range: 0–300). The semi-automated image analysis workflow is graphically depicted in Figure S2G.

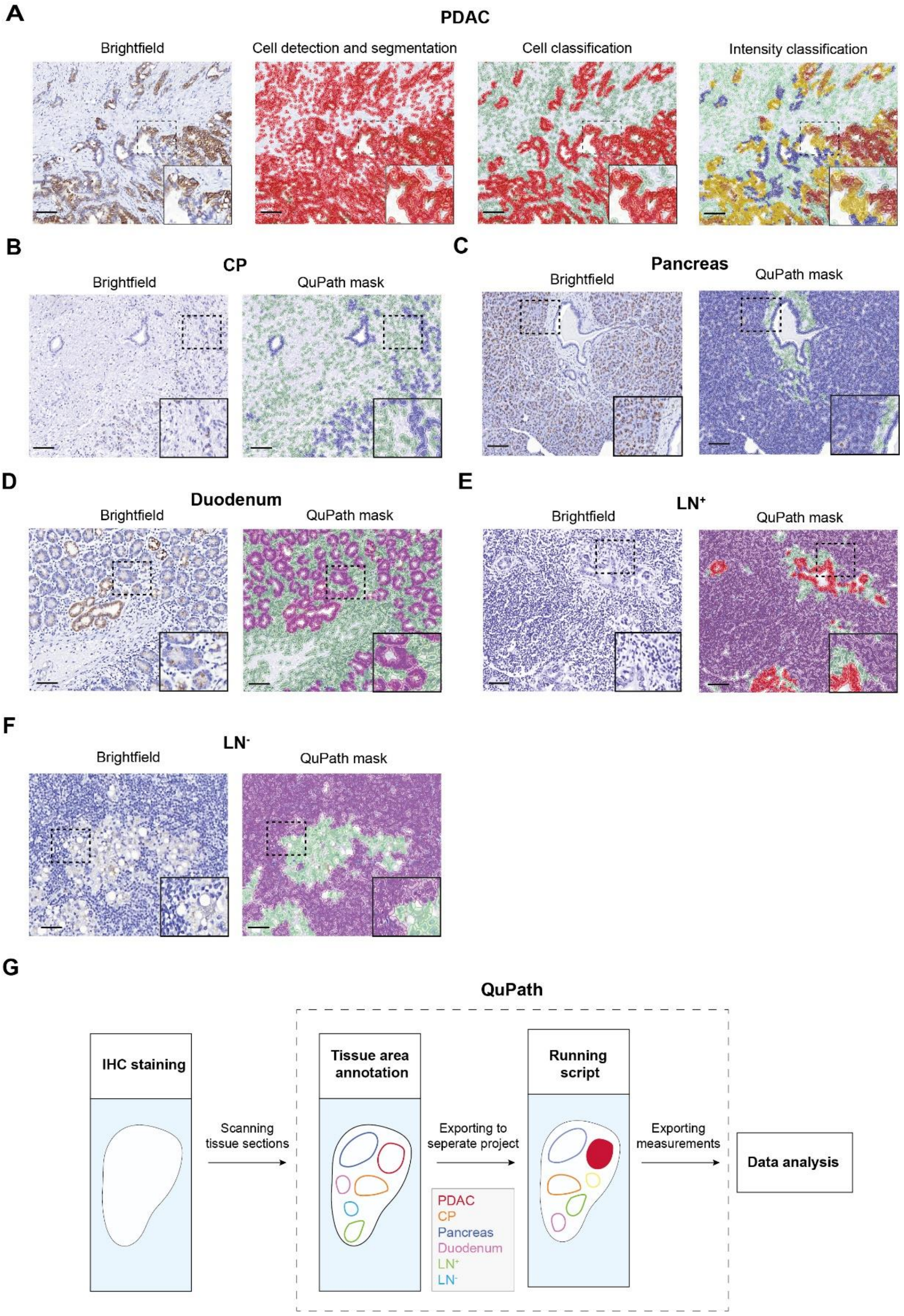


**Figure S2**. QuPath images and semi-automated image analysis workflow. (**A**) Example of brightfield, cell detection and segmentation, cell classification and intensity classification images of a PDAC tissue area in QuPath; **(B–F)** representative brightfield and QuPath mask images of CP, pancreas, duodenum, LN+ and LN− tissues before after application of the object classifier. Brown: DAB, Red: tumor cells, green: stromal cells, blue: acinar cells, pink: duodenal gland cells, purple: immune cells. Overview images and inserts are taken at 5× and 25× magnification, respectively. Scale bars represent 100 µm; **(G)** semi-automated image analysis workflow. Tissue sections were scanned and imported into QuPath. Thereafter, tissue areas were annotated and files were exported to tissue class-specific projects in which the biomarker-, tissue class-

specific script was run. Next, cell measurements were exported and data were analyzed. CP: chronic pancreatitis, IHC: immunohistochemistry, LN+: positive lymph node, LN−: negative lymph node, PDAC: pancreatic ductal adenocarcinoma.

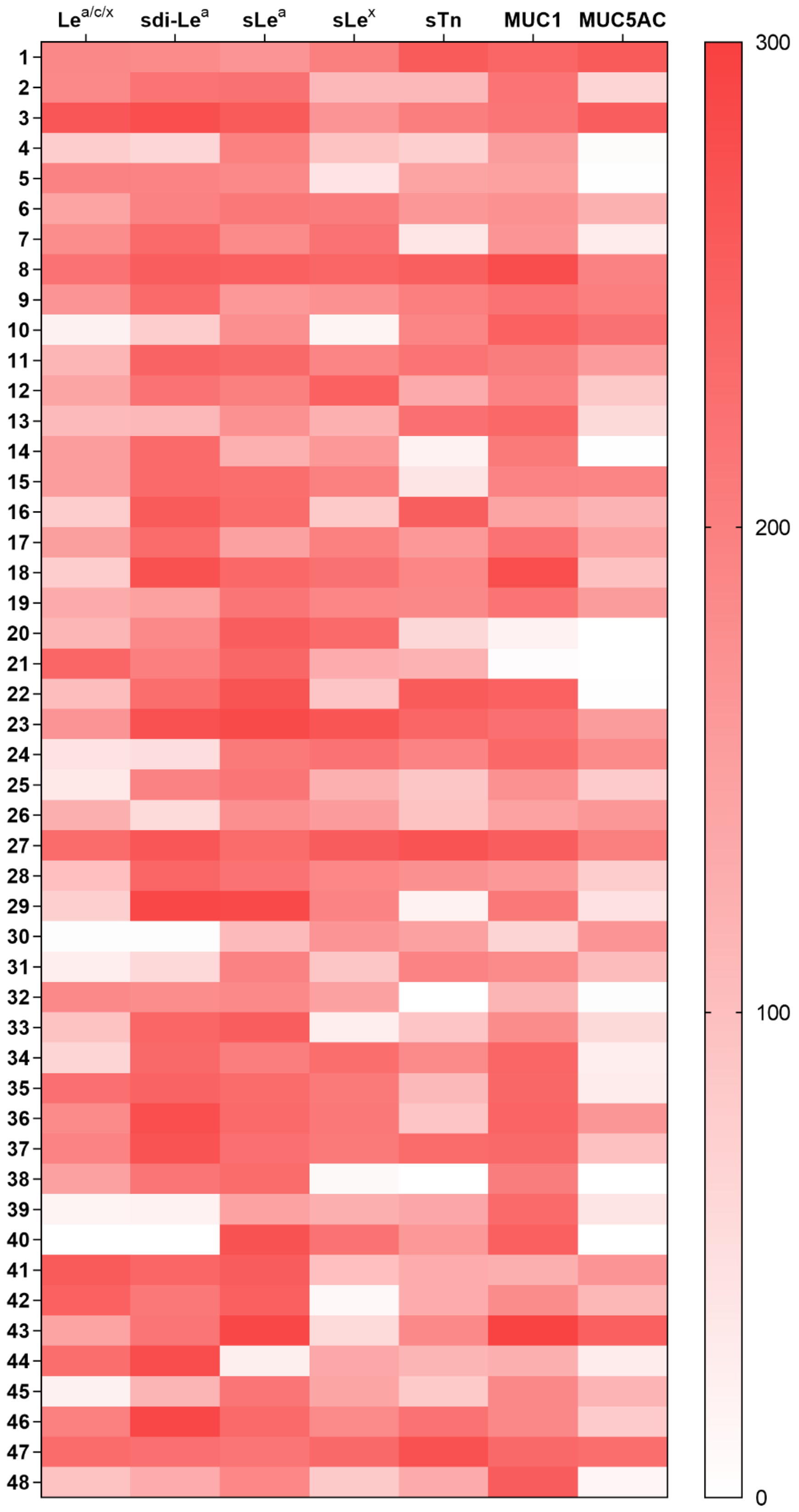


**Figure S3.** Heatmap of biomarker expression on PDAC tissues for each case separately. The color bar indicates which red color tint corresponds to which H-score.

**Table S1.** Primary and secondary mAbs, clone, catalog number, provider, isotype and conditions used during IHC. Conc: concentration.

| Biomarker | Clone | Catalog Number | Provider | Isotype | Antigen Retrieval | Conc. (μg/mL) |
|---|---|---|---|---|---|---|
| | | | *Primary Antibodies* | | | |
| $Le^{a/c/x}$ | FG88.2 | - | Developed by Scancell Ltd. and kindly provided by professor Lindy Durrant | Mouse $IgG_3$ | Heating for 10 min at 95 °C in EnVision FLEX™ Target Retrieval Solution, Low pH (K8005, Agilent) using PT Link (Agilent) | 0.19 |
| sdi-$Le^a$ | FG129 | - | Developed by Scancell Ltd. and kindly provided by professor Lindy Durrant | Mouse $IgG_{1}κ$ | Heating for 10 min at 95 °C in K8005, Agilent using PT Link (Agilent) | 0.12 |
| $sLe^a$ | 121SLE | Ab3982 | Abcam, Cambridge, UK | Mouse IgM | Heating for 10 min at 95 °C in EnVision FLEX™ Target Retrieval Solution, High pH (K8004, Agilent) using PT Link (Agilent) | 0.16 |
| $sLe^x$ | CSLEX1 | 551344 | BD Pharmingen™, Franklin Lakes, NJ, USA | Mouse IgM, κ | Heating for 10 min at 95 °C in K8005, Agilent using PT Link (Agilent) | 0.55 |
| sTn | CC49 | - | Antibodies for Research Applications BV, Gouda, the Netherlands | Mouse $IgG_{1}κ$ | - | 2.0 |
| MUC1 | E29 | MA5-14077 | ThermoFisher, Waltham, MA, USA | Mouse $IgG2_a$ | Heating for 10 min at 95 °C in K8005, Agilent using PT Link (Agilent) | 0.03 |
| MUC5AC | CLH2 | sc-33667 | Santa Cruz Biotechnology, Inc., Dallas, USA | Mouse $IgG_{1}κ$ | Heating for 10 min at 95 °C in K8005, Agilent using PT Link (Agilent) | 0.5 |
| | | | *Secondary Antibody* | | | |
| Anti-mouse | - | K4001 | Agilent Technologies, Santa Clare, CA, USA | Goat antimouse IgG | - | Stock concentration |

**Table S2.** Automated cell detection parameters used in QuPath.

| Parameter | Value |
|---|---|
| Setup parameters | |
| Request pixel size | 0.3890 μm |
| Nucleus parameters | |

| | |
|---|---|
| Background radius | 8 µm |
| Median filter radius | 0 µm |
| Sigma | 2.0 µm |
| Minimum area | 10 µm$^2$ |
| Maximum area | 400 µm$^2$ |
| Intensity parameters | |
| Threshold | 0.1 |
| Max background intensity | 2.0 |
| Split by shape | True |
| Exclude DAB (membrane staining) | True |
| Cell parameters | |
| Cell expansion | 5 µm |
| Include cell nucleus | True |
| General parameters | |
| Smooth boundaries | True |
| Make measurements | True |

**Table S3.** Mean ± SD object classifier sensitivity, specificity, PPV, NPV and accuracy for the detection of tumor, stromal, acinar, immune or glandular cells from all biomarkers combined ($n$ = 7), as described in supplementary materials. LN+: positive lymph node, LN−: negative lymph node, PPV: positive predictive value, NPV: negative predictive value, Sens: sensitivity, Spec: specificity.

| Tissue Class | Detection | Sens. | Spec. | PPV | NPV | Accuracy |
|---|---|---|---|---|---|---|
| Tumor | Tumor vs. stromal cells | 91.5 ± 1.4 | 99.0 ± 0.5 | 98.7 ± 0.6 | 92.5 ± 2.0 | 95.4 ± 0.7 |
| Pancreas | Acinar vs. stromal cells | 94.9 ± 2.8 | 96.6 ± 1.9 | 97.7 ± 1.5 | 93.0 ± 2.8 | 95.9 ± 1.6 |
| Duodenum | Glandular vs. stromal cells | 94.6 ± 3.9 | 89.7 ± 2.9 | 91.0 ± 2.5 | 93.8 ± 4.2 | 95.7 ± 2.4 |
| LN+ | Tumor vs. stromal + immune cells | 90.5 ± 4.5 | 97.3 ± 1.6 | 92.7 ± 3.6 | 96.7 ± 2.2 | 97.1 ± 2.1 |
| LN− | Immune + stromal cells | 97.6 ± 2.4 | 94.9 ± 2.4 | 98.5 ± 0.8 | 92.8 ± 5.8 | 92.2 ± 1.8 |
